# Data Access, User Engagement, and Interoperability Standards in Public Health Data Portals: Environmental Scan Study

**DOI:** 10.2196/95523

**Published:** 2026-10-07

**Authors:** Yogita Chaudhary, Lathika R, Solomon Thirumurugan, Manikandan Kumaraguru, Sharan Murali, Rizwan Suliankatchi Abdulkader

**Affiliations:** 1 Intramural Research Team, ICMR National Institute of Epidemiology, Chennai, TN, India; 2 ICMR National Institute of Epidemiology, 2nd Main Rd, near Ambattur, Karmari Amman Nagar, TNHB Colony, Bhavani Nagar, Ayappakkam, Chennai, TN, India, 91 8903307313

**Keywords:** standards, health information system interoperability, digital health, Systematized Nomenclature of Medicine Clinical Terms, SNOMED CT, health information exchange

## Abstract

**Background:**

Rapid digitalization has led to the generation of vast volumes of health data across diverse sources, including public health surveillance, electronic health records from hospitals, primary care facilities, and telemedicine. However, it often remains fragmented across multiple domains and systems due to siloed data collection practices and a lack of interoperability standards, limiting its potential to generate actionable public health insights.

**Objective:**

In this study, we examined publicly available health data portals and assessed their scope, user engagement features, and implementation of interoperability standards.

**Methods:**

We conducted an environmental scan to profile publicly available health data portals. We identified portals through systematic web searches, expert consultation using the Delphi technique, and snowball sampling from initial sources. We assessed each portal for the type of data hosted, the scope of user engagement, and the implementation of interoperability standards (Systematized Nomenclature of Medicine Clinical Terms [SNOMED CT]) or adopted Fast Healthcare Interoperability Resources (FHIR) as a data exchange standard.

**Results:**

We identified 17 data portals in total, of which 9 (53%) hosted global data and 1 (6%) hosted regional data. Portals hosted data across diverse domains including communicable diseases and noncommunicable diseases, maternal and child health, injuries, and others. Most portals supported basic user interactions such as data querying (n=15, 88%) and downloading (n=15, 88%), but none offered in-portal analytics. While 3 (18%) portals used *International Classification of Diseases* (*ICD*) coding systems, none implemented SNOMED CT or adopted FHIR.

**Conclusions:**

Our study highlighted substantial fragmentation and a lack of interoperability across health data portals, which limits the ability to conduct integrated analysis and obtain comprehensive public health insights. Realizing the full potential of health data will require accessibility and interoperability, intelligent analytics, and seamless integration across domains.

## Introduction

The emphasis on digital health has grown tremendously worldwide in recent years, driven by advances in information and communication technology (ICT) aimed at improving health outcomes [1]. ICT is increasingly harnessed to promote health, prevent diseases, and improve service delivery. A vast amount of digital health care data are generated across domains such as public health surveillance and disease outbreaks, electronic health records (EHRs) from hospitals and primary care settings, and telemedicine, which are lying fragmented and siloed [2-6].

Fragmented data systems pose a major barrier to fully leveraging existing data sources to generate actionable knowledge that can be translated into practice [7]. There is a lack of adherence to the 4 principles of data management—findability, accessibility, interoperability, and reusability (FAIR)—which limits effective data exchange and its use [8]. Addressing these challenges requires innovative approaches.

Big data solutions, which aim to extract knowledge and insights from large and complex digital data, offer promising opportunities. They can integrate the silos to generate insights for better epidemiology surveillance, research, and efficient health care delivery [2]. Clinical vocabularies, data standards, and interoperability are an integral part of big data solutions and effective digital health strategies [2,9]. Interoperable systems enable accurate data collection and sharing across countries and organizations without information loss [1,9,10].

Systematized Nomenclature of Medicine Clinical Terms (SNOMED CT) and Health Level 7 (HL7) Fast Healthcare Interoperability Resources (FHIR) are among the top recommended standards to achieve interoperability [10-12]. SNOMED CT is a formally structured, consensually developed clinical terminology that provides standardized representations of clinical and public health concepts, enabling semantic interoperability across systems. SNOMED CT is the most comprehensive, multilingual clinical health care terminology globally, with more than 360,000 concepts. It has three core components: (1) concepts—represent a unique clinical term; (2) relationships—define the association between two concepts; and (3) descriptions—provide human-readable terms to convey the meaning of the clinical term [13]. SNOMED CT enables the consistent encoding of clinical and public health data, helping researchers worldwide better understand regional data and integrate them with other datasets [14,15].

HL7 FHIR, by contrast, is a data exchange standard that defines how health-related information is structured and transmitted between systems. HL7 FHIR standards were developed by the HL7 organization in 2011. It has two primary components: (1) resources—determine the structure and elements of exchangeable data—and (2) APIs—enable interoperability between the applications. It supports various health care domains through resources such as “patient,” “procedure,” and “observation,” making it adaptable for different purposes and contexts [16].

In recent years, an increasing number of countries have adopted HL7 FHIR and SNOMED CT standards to implement interoperability frameworks [10,17,18]. These standards are commonly used for EHRs in the health system [10]. However, their adoption is limited in the public health system [19]. We conducted an environmental scan of publicly accessible digital health data portals to describe the variety of health data hosted and their scope of user engagement. We also evaluated the implementation of HL7 FHIR and SNOMED CT standards in these portals.

## Methods

An environmental scan of digital health data portals was conducted between December 2023 and May 2024.

### Search Strategy

We used the following methods to profile digital portals that host health databases:

1. Search engines: we used Google as our primary search engine, using keywords such as “online healthcare portals,” “online healthcare data portals,” “digital health portals,” “healthcare data portals,” “health data portals,” “data portals,” “public health,” and “population level data” both in combinations and separately.
2. Delphi technique: we consulted scientists and epidemiologists at the Indian Council of Medical Research (ICMR), National Institute of Epidemiology (NIE), Chennai, to identify health-related data portals of which they were aware.
3. Snowballing from initial sources: we identified additional relevant portals by snowball sampling from the portals identified through methods 1 and 2.

### Inclusion Criteria

We included digital portals that store any type of health-related data, are publicly accessible, and are available free of charge. We excluded portals whose primary language was not English.

### Operational Definition

We used the following operational definitions throughout our study:

- Data analysis (also referred to as in-portal analysis): data analysis refers to users’ ability to perform computational or statistical operations on raw data within the portal, including deriving new variables and examining relationships between the existing ones.
- Data granularity: data granularity refers to the level at which data are collected, stored, and presented [20]. The granularity of data hosted is categorized into 3 levels: global, country, or regional.
- Download data: downloading data refers to the ability of users to download datasets of interest from the portal in any format for offline use.
- Health data portals: health data portals are web-based interfaces that organize and present health-related data from various sources for discovery and efficient use by policymakers, researchers, journalists, and the public [21].
- Query the data: querying, in the context of database management, refers to the process of requesting specific data and retrieving the information as a table or graph, based on preaggregated, fixed indicators [22].
- Upload data: uploading data refers to the ability of users to contribute their own datasets to the portal for data sharing and collaborative use, as well as to perform computational or statistical operations on the uploaded data within the portal.
- Ontologies: we defined ontology as a formally structured, consensually developed clinical terminologies and coding systems that provide standardized representations of clinical and public health concepts.

### Data Extraction and Analysis

We reviewed the included portals and extracted their aims, visions, missions, ownership statuses, data granularity, types of health data hosted, scopes of user engagement, and adoption of SNOMED CT as a clinical terminology and HL7 FHIR as a data exchange standard onto an Excel spreadsheet. We summarized the characteristics of the portals in a table and visualized the types of health data hosted, user engagement scope, and adoption of standards in an infographic. Given the Indian research context of this study, we described the Indian digital health data portals as case studies to provide deeper insights into their capabilities and limitations. From the list of non-Indian portals, we selected 3 portals that represented a diversity of ownership, including an intergovernmental body, a government agency, and a collaborative nonprofit initiative.

We categorized the health data hosted in the study portals into five domains: (1) communicable disease (CD); (2) noncommunicable disease (NCD); (3) injuries; (4) reproductive, maternal, newborn, child, and adolescent health and nutrition (RMCH+N); and (5) others. We included data that did not fit into the primary categories in the “others” category.

The scope of user engagement with the portal was classified at 4 levels—the ability of a user to query the data, upload data, analyze data, and download data. We summarized the findings on portal ownership, level of data granularity, data domains, scope of user engagement, and data standards as frequencies and proportions.

### Ethical Considerations

This study is part of a larger ICMR-NIE project titled “ICMR Epihub: A platform for epidemic intelligence and interpretation of infectious disease epidemiology.” This study has been approved by the Institutional Human Ethics Committee (IHEC) of ICMR-NIE (NIE/IHEC/202210‐03). Personal information was not included in this study; thus, informed consent was not required.

## Results

### Overview

A total of 18 portals met the inclusion criteria, of which 1 portal was excluded because its primary language was not English (Figure 1). The overarching objective of these portals was to enhance the accessibility of health care data, thereby improving the understanding of health outcomes for individuals and nations (Table 1).

**Figure 1.**
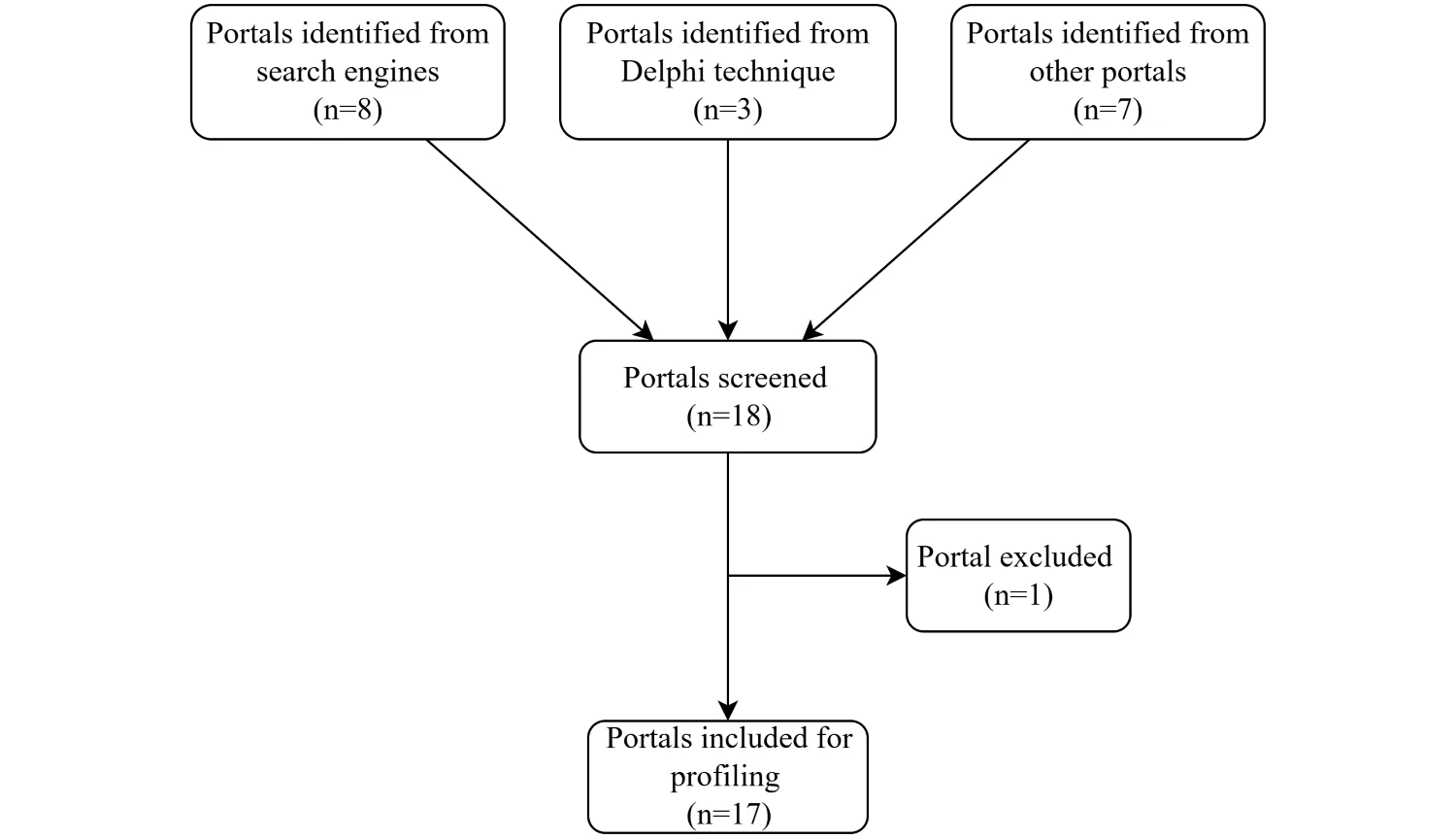
Health data portals included in the analysis.

**Table 1.** Overview of digital health portals: objectives, ownership, and data granularity.

Portal name	Ownership	Data granularity
Centers for Disease Control and Prevention Wide-Ranging Online Data for Epidemiologic Research [22]	Government (US Department of Health and Human Services)	Country (United States)
Gapminder [23]	Nonprofit (Gapminder Foundation)	Global
Health Indicators Warehouse [24]	Government (US Department of Health and Human Services)	Country (United States)
Institute for Health Metrics and Evaluation portal [25]	Academic (IHME^a^, University of Washington)	Global
India Policy Insights [26]	Collaborative (Geographic Insights Lab, IIPS^b^, and NITI^c^ Aayog)	Country (India)
National Data and Analytics Platform [27]	Government (NITI Aayog)	Country (India)
Noncommunicable Disease Risk Factor Collaboration [28]	Collaborative (WHO^d^ and academic partners)	Global
National Center for Health Statistics Data Query System [29]	Government (US Department of Health and Human Services)	Country (United States)
Organisation for Economic Co-operation and Development Data Explorer [30]	Intergovernmental (OECD^e^)	Country (OECD+selected non-OECD countries)
Our World in Data [31]	Collaborative (Global Change Data Lab and University of Oxford)	Global
Rochester Epidemiology Project data exploration portal [32]	Collaborative (County of Olmsted, Mayo Clinic, Mayo Clinic Health System, Olmsted Medical Center, and Zumbro Valley Health Center)	Regional
State of Global Air [33]	Collaborative (Health Effects Institute and IHME)	Global
The Global Health Observatory [34]	UN^f^ agency (WHO)	Global
The Socioeconomic High-Resolution Rural-Urban Geographic [35]	Nonprofit (Development Data Lab)	Country (India)
United Nations Inter-agency Group for Child Mortality Estimation [36]	Collaborative (UNICEF^g^, UN DESA^h^, WHO, and World Bank)	Global
United Nations Population Division Data Portal [37]	UN agency (UN DESA)	Global
United Nations Sustainable Development Goals Indicators database [38]	UN agency (UN DESA)	Global

a IHME: Institute for Health Metrics and Evaluation.

b IIPS: International Institute for Population Sciences.

c NITI: National Institution for Transforming India.

d WHO: World Health Organization.

e OECD: Organisation for Economic Co-operation and Development.

f UN: United Nations.

g UNICEF: United Nations Children's Fund.

h UN DESA: United Nations Department of Economic and Social Affairs.

### Ownership

Of the 17 health data portals, governments own 4 (24%) portals, nonprofit organizations own 2 (12%) portals, and 6 (35%) portals are managed in collaboration. The remaining 5 (29%) portals are maintained by academic institutions, intergovernmental bodies, or United Nations agencies (Table 1).

Among the 4 government-owned portals, the US Department of Health and Human Services manages 3 (75%) portals, namely, the National Center for Health Statistics Data Query System, Health Indicators Warehouse, and Centers for Disease Control and Prevention Wide-Ranging Online Data for Epidemiologic Research (CDC Wonder) [22,24,29]. In India, the National Institution for Transforming India Aayog manages the National Data and Analytics Platform (NDAP) [27].

### Level of Data Granularity

Of the 17 portals, 9 (53%) provided health estimates at the global level, 7 (41%) reported data at the country level, while 1 (6%) reported data at the regional level (Table 1). Among the country-level portals, 3 portals focus exclusively on India-specific data, namely, NDAP, India Policy Insights, and Socioeconomic High-Resolution Rural-Urban Geographic (SHRUG) [26,27,35]. Three portals focus exclusively on US-specific data, and the Organisation for Economic Co-operation and Development (OECD) Data Explorer focuses on OECD and selected non-OECD countries [22,24,29,30].

### Domains Covered by the Data

A total of 11 (65%) portals hosted data related to CD, 12 (71%) had data on NCD, 12 (71%) had data on RMCH+N; 8 (47%) portals hosted data related to injuries, and 7 (41%) portals, namely, CDC Wonder, Gapminder, Health Indicator Warehouse, Institute for Health Metrics and Evaluation (IHME) portal, OECD Data Explorer, the Global Health Observatory (GHO), and United Nations Sustainable Development Goals Indicators database, provided extensive data on all 5 categories (Figure 2) [22-25,30,34,38]. The “other” category includes data related to health behavior; health economics and policy; demography and population; environment and pollution; health systems and infrastructure; pharmaceutical markets; and specialized areas such as oral health, antimicrobial resistance, and the sustainable development goals (SDGs).

**Figure 2.**
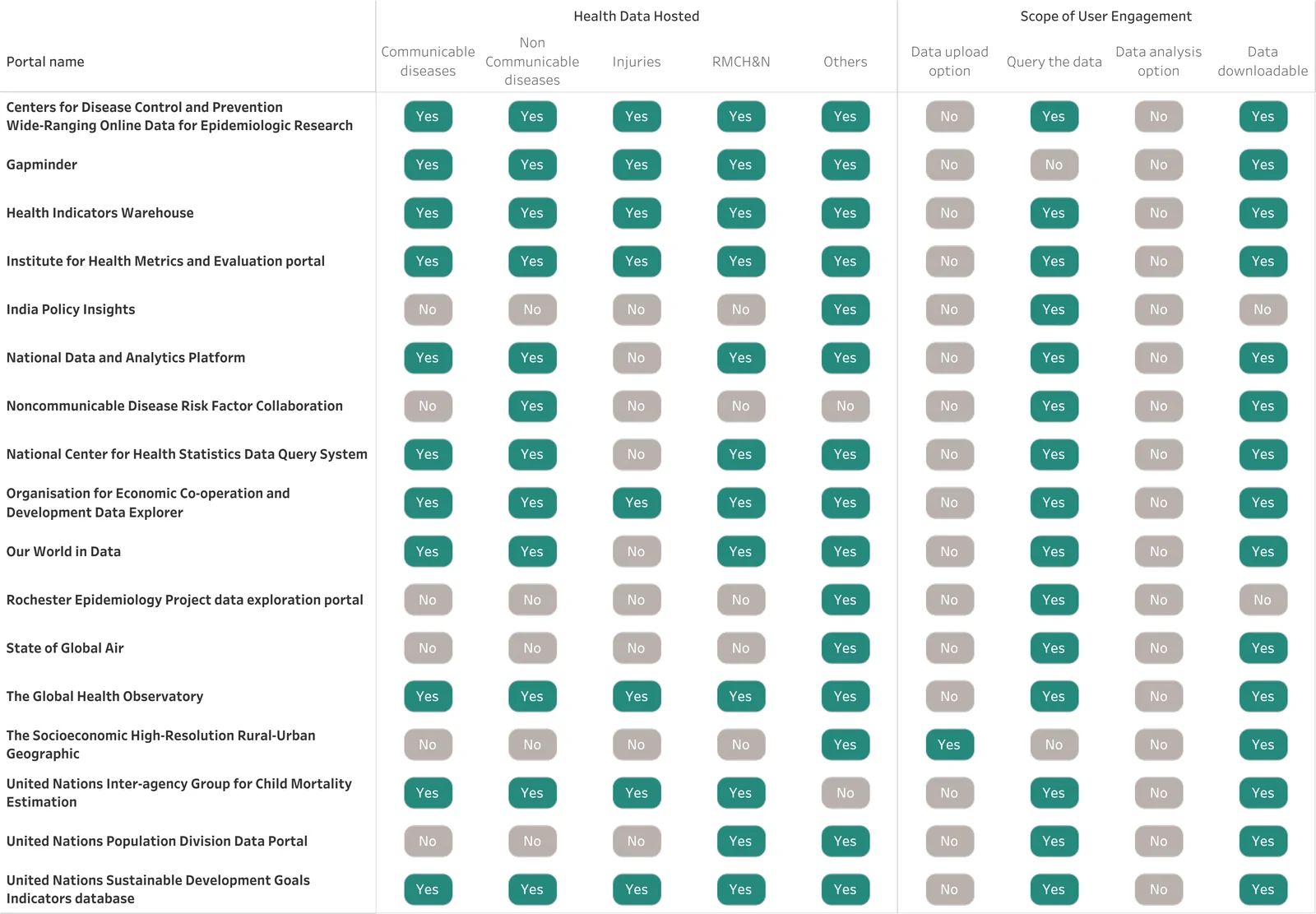
Insights into data hosted and the scope of user engagement in the portals reviewed. ..CDC Wonder: Centers for Disease Control and Prevention Wide-Ranging Online Data for Epidemiologic Research; IHME: Institute for Health Metrics and Evaluation; NCD: noncommunicable disease; NCHS: National Center for Health Statistics; OECD: Organisation for Economic Co-operation and Development; RMCH+N: reproductive, maternal, newborn, child and adolescent health and nutrition; SDG: sustainable development goal; SHRUG: Socioeconomic High-Resolution Rural-Urban Geographic; UN: United Nations.

### Scope of User Engagement

The scope of user engagement varied across the portals reviewed. Of the 17 portals, 15 (88%) allowed users to query the data directly, except for Gapminder and SHRUG, which did not support data querying [23,35]. Similarly, 15 (88%) portals, except India Policy Insights and the Rochester Epidemiology Project (REP) data exploration portal, supported data downloads [26,32]. The India Policy Insights portal helps visualize queried data as maps; only these maps are downloadable [26]. The REP data exploration portal does not allow users to download the data from their database in any form [32]. Only 1 (6%) portal, the SHRUG, permitted users to upload their data as a contribution to their database [35]. None of the portals had in-portal analytics capabilities (Figure 2).

### Data Standards Used

Of the 17 portals, 3 (18%), namely CDC Wonder, IHME, and GHO, stored the data using *International Classification of Diseases* (*ICD*) codes [22,25,34]. While a few portals, notably the GHO, supported programmatic data access through APIs, none adopted an FHIR for structured data exchange. This pattern was consistent across both Indian and international portals, suggesting that API-based access, when available, primarily serves as a convenience tool for data retrieval rather than as a mechanism for semantic interoperability.

### Selected Non-Indian Portals

The GHO, managed by the World Health Organization (WHO), provided access to more than 1000 indicators on priority health topics across its 194 member states, with a vision to promote health, keep the world safe, and serve the vulnerable [34]. It hosts data spanning CDs and NCDs, maternal and child health, health systems, and the SDGs, drawn from household surveys, civil registration systems, and facility-based sources. Users can query and download data across all domains. Notably, the GHO is the only portal reviewed to support standardized programmatic data access via an OData-compliant API, enabling users and software applications to retrieve datasets in a consistent and predictable way. Health indicators are also being transitioned to universally unique identifiers to strengthen long-term interoperability across datasets and partner systems.

CDC Wonder, managed by the US Department of Health and Human Services, provides access to a wide range of public health data for the United States, spanning CDs and NCDs, mortality, natality, and environmental health [22]. It is 1 of 3 portals that encodes data using the *ICD* system, providing a degree of semantic standardization for cause-of-death and disease classification. Users can query and download data, and the portal supports programmatic access through a proprietary XML-based API.

Our World in Data (OWID), a collaborative initiative of the Global Change Data Lab and the University of Oxford, aims to make data on the world’s largest problems accessible to a broad audience [31]. It hosts data spanning health, poverty, education, climate, and demography, drawn from international sources including the WHO, the World Bank, and academic institutions. Users can query, visualize, and download data. More recently, the portal introduced a Chart Data API enabling access to datasets. OWID aggregated data from diverse original sources with differing coding conventions. There is no harmonization of health concepts across datasets, which limits their utility for cross-dataset analytical work.

### Indian Portals

NDAP is developed with a vision to make data generated by the government accessible to the public [27]. All survey data spanning 31 sectors and 53 ministries are available as aggregates for the fixed indicators. All datasets are standardized to a common schema and mapped to a unified geographical framework using the Ministry of Panchayati Raj Local Government Directory Code, enabling users to merge datasets across sectors and sources for cross-sectoral analysis. This portal enables users to create queries based on predefined indicators for each dataset and download the data and results. Notably, NDAP represents one of the more deliberate efforts among the reviewed portals to address interoperability not through health-specific standards such as the FHIR or SNOMED CT but through a common administrative schema that enables linkage across government datasets.

The India Policy Insights portal informs evidence-based health policy decisions through its online geo-visual data portal [26]. The portal aggregated data from various sources, including population censuses, health surveys, the COVID-19 vaccination dashboard (COWIN), Datameet, assembly constituencies, and parliamentary constituencies [39]. It tracks the impact of health policies through an interactive dashboard through various use cases such as the National Family Health Survey Policy Tracker for Parliamentary Constituencies, India COVID-19 Vaccine Tracker, and Visualizing Child Undernutrition across Assembly Constituencies in India. Users can only interact with the data through these fixed dashboards, and there is no option to download the data. The portal does not adopt any recognized data exchange standard, and its fixed dashboard structure limits user engagement to passive visualization rather than active data exploration.

The SHRUG is a research collaboration portal that facilitates data upload and download and was developed by the Development Data Lab, a nonprofit organization [35]. This portal harnesses data from various sources, including population census, socioeconomic caste census, and geospatial data for India. SHRUG also hosts data from special projects, such as Mission Antyodaya Village Facilities and Facebook Wealth and Population statistics. It has generated specific use cases, such as optimal locations for decentralized health care delivery, changes in population characteristics with proximity to towns, and dynamic changes in socioeconomic class structure in India. A distinctive feature of SHRUG is its use of a consistent set of universal geographic identifiers, the shrid, which links all datasets at the village and town level, enabling researchers to merge data from disparate sources without additional harmonization effort. However, similar to the other portals reviewed, SHRUG does not adopt health-specific interoperability standards and does not allow the user to query the data or perform any in-portal analysis.

## Discussion

### Principal Findings

We reviewed the publicly available health data portals to profile them and identify the scope of user engagement and the use of standard ontologies. We identified 17 portals that enhanced access to health data across domains and geographies. These portals have democratized access to health-related information and empowered users, ranging from policymakers to journalists, to engage with data and insights more readily. However, they lacked capabilities for dynamic user-driven analysis and real-time insights for alerts or clinical decision support. We also identified that none of these platforms used interoperable standards such as SNOMED CT and HL7 FHIR.

Our study identified that health care datasets are publicly available for download and use. We believe this has significantly enhanced access to health-related information, empowering policymakers and researchers to engage with health data more readily and use it to inform their work. Although the public is unlikely to query or download raw data directly, they typically rely on summary dashboards, visualizations, and narrative reports to interpret health information. The availability of raw, quarriable data nonetheless remains valuable for public health practitioners and other technical users within the health domain, who are better positioned to derive actionable insights from it. Overall, these portals have democratized data access and facilitated transparency and informed decision-making in public health [40,41]. However, we identified that the capacity for users to perform in-depth analyses within these portals remains limited. If these portals do not provide tools to analyze the data or generate insights directly, the responsibility of deriving meaning from the data falls back onto the user and their skills. This can hinder the real-time translation of the data into actionable public health interventions.

We identified that the health data portals are characterized by fragmentation, where disparate datasets are scattered across various portals, each serving distinct purposes. This disjointed approach hampers the ability to perform integrated analyses essential for comprehensive public health insights [42]. For instance, although rainfall data may be available through meteorological departments and malaria incidence data through health surveillance systems, the lack of interoperability between these datasets prevents researchers from effectively correlating climatic factors with disease outbreaks. This disconnect restricts the public health community’s capacity to conduct predictive analyses or design targeted interventions [43].

We identified that none of the portals used interoperable standards such as the SNOMED CT and HL7 FHIR. The literature from low- and middle-income countries also highlighted similar challenges, where health data systems operate in silos due to the absence of common metadata schemas, harmonized ontologies, or standardized data-sharing protocols [42,44]. During the COVID-19 pandemic, data standards and infrastructure made it difficult to harness the power of AI and predictive data science to improve clinical care for patients with COVID-19 [11]. The COVID-19 pandemic highlighted the necessity of globally aligned standardization of health care data to respond to future public health crises [11,42]. Without integration, data remain underused, and opportunities for system-wide learning are lost. Interoperable systems can enhance routine and emergency public health activities, such as surveillance and outbreak management, by enabling faster responses and earlier interventions [6]. Thus, there is a need to develop portals that aggregate datasets from diverse sectors using data exchange standards such as HL7 FHIR.

Health data portals should also support layered analysis across domains such as health, climate, demographics, and socioeconomics, empowering stakeholders to act on evidence in real time [42,45]. This will help democratize access to data and facilitate their use for evidence-based action in the field. We must leverage the current digital era and the evolving capabilities of AI to harness the true power of health data. When paired with interoperable frameworks such as the SNOMED CT and HL7 FHIR, they can transform unstructured data into standardized formats, enabling predictive analytics and intelligent decision support systems [46]. Leveraging AI in this way not only supports scalable public health surveillance but also accelerates the development of responsive, data-driven health systems suited for both routine and emergency settings.

Although we performed this analysis in the timely context of postpandemic digital health reforms, our study had some limitations too. First, the search strategy used in this study, which relied on web searches, expert consultation, and reference snowballing, was not exhaustive and may have resulted in the omission of eligible portals, particularly those from non–English-speaking countries or those less prominently indexed online. Second, the assessment of user engagement relied on interface reviews and publicly available documentation. We have not performed any formal testing on or collected empirical user feedback from these portals. This may limit the generalizability of conclusions on user engagement.

Third, the capabilities of these portals were not scored using a standardized appraisal framework, which may introduce some subjectivity in comparisons. Fourth, given the dynamic nature of digital health portals, the findings represent a snapshot in time and may not reflect recent updates or feature enhancements. Finally, the study focused solely on publicly accessible portals and did not include closed systems or internal dashboards used within ministries or large health programs, which may offer richer analytical capabilities. It is important to note that such systems are intentionally restricted from public access, as the depth of data and analytical functionality they provide is neither required nor appropriate for the general public. These internal systems might host more granular data and support more advanced analytical capabilities than those observed in the publicly accessible portals reviewed here. Consequently, the findings and conclusions of this study should be interpreted strictly within the context of publicly accessible health data infrastructure and should not be generalized to the broader ecosystem of health information systems.

### Conclusions

Our study highlights the need for health data portals to move beyond data access toward interoperability and richer in-portal analytical capabilities. Addressing fragmentation and limited user functionality is essential to support actionable, equity-focused insights. Future health data portals should adopt standard ontologies and analytical tools to empower diverse users across sectors.

## Abbreviations

CD: communicable disease
CDC Wonder: Centers for Disease Control and Prevention Wide-Ranging Online Data for Epidemiologic Research
EHR: electronic health record
FAIR: findability, accessibility, interoperability, and reusability
FHIR: Fast Healthcare Interoperability Resources
GHO: Global Health Observatory
HL7: Health Level 7
*ICD*: *International Classification of Diseases*
ICMR: Indian Council of Medical Research
ICT: information and communication technology
IHEC: Institutional Human Ethics Committee
IHME: Institute for Health Metrics and Evaluation
NCD: noncommunicable disease
NDAP: National Data and Analytics Platform
NIE: National Institute of Epidemiology
OECD: Organisation for Economic Co-operation and Development
OWID: Our World in Data
REP: Rochester Epidemiology Project
RMCH+N: reproductive, maternal, newborn, child and adolescent health and nutrition
SDG: sustainable development goal
SHRUG: Socioeconomic High-Resolution Rural-Urban Geographic
SNOMED CT: Systematized Nomenclature of Medicine Clinical Terms
WHO: World Health Organization
